# Non-operative Treatment of Mason Type I Radial Head Fractures: A Comparative Analysis Using Patient-Reported Outcomes Measurement Information System (PROMIS)

**DOI:** 10.7759/cureus.42056

**Published:** 2023-07-18

**Authors:** Thomas J Carroll, Akhil Dondapati, Jordan Cruse, Jonathan Minto, Warren C Hammert, Bilal Mahmood

**Affiliations:** 1 Orthopaedic Surgery, University of Rochester, Rochester, USA; 2 Orthopaedic Surgery, Duke University, Durham, USA

**Keywords:** promis scores, radial head, radial head instability, radial head arthroplasty, radial head fracture

## Abstract

Objectives: The purpose of this study is to compare the outcomes of Mason type I radial head fractures. This information will help to provide physicians with a critical decision-making tool when considering non-operative intervention and evaluate Patient-Reported Outcomes Measurement Information System (PROMIS) as a potentially valuable measure to track outcomes.

Methods: We retrospectively identified 527 patients undergoing non-operative intervention. Demographic information, physical exam measurements, patient acceptable symptom state (PASS), and PROMIS Upper Extremity (UE), Physical Function (PF), and Pain Interference (PI) scores were analyzed over 12 months.

Results: At the initial outpatient post-injury visit (within one week of injury), the average PROMIS PF, UE, PI, and Depression were 42.04 (SD: 6.3), 35.31 (SD: 7.3), 59.18 (SD: 9.2), and 48.68 (SD: 6.8), respectively. The average change in PROMIS PF, UE, PI, and Depression scores from the time of injury to six weeks were -0.23 (p=0.7), 1.43 (p=0.03), -2.1 (p=0.01), and -0.99 (p=0.1). The average change in PROMIS PF, UE, PI, and Depression scores from the time of injury to six months was -0.56 (p=0.56), 1.84 (p<0.001), -1.84 (p<0.001), and -0.13 (p=0.68). Among patients initially reporting “not acceptable” on PASS and reporting “acceptable” at the six-month visit, the average PROMIS PF, UE, PI, and Depression scores were 42.14, 38.91, 56.91, and 47.51 respectively. This represents an average difference of 1.11 (p=0.07), 2.82 (p<0.01), -1.19 (p=0.04), and -1.7 (p=0.01) respectively.

Conclusion: PROMIS UE and PI significantly improved among Mason I radial head fractures treated non-operatively at both six-week and six-month follow-up points but did not meet the mean clinically important difference (MCID) PROMIS PF did not significantly differ between the time of injury, six-week or six-month follow-up points. Only PROMIS UE correlated with PASS at six-week and six-month follow-up. Among patients who improved from negative to positive responses on PASS, PROMIS UE, and PI significantly improved.

## Introduction

Radial head fractures are the most common type of fractures, about the elbow, making up nearly one-third of all elbow fractures and have an annual incidence of around two to three per 10,000 people [1,2]. In addition to its role in pronation and supination, the radial head is also an important stabilizer of the elbow as it serves as a secondary valgus stabilizer and provides longitudinal stability [3]. Radial head fractures are commonly characterized using the Mason classification system, which helps describe the extent of displacement and the degree of radial head involved. Mason type I fractures are non-displaced or have <2mm of displacement with no mechanical blocks to motion. Type II fractures involve part of the articular surface or have >2mm displacement. Type III are comminuted, displaced fractures involving the entire radial head. Type IV fractures denote radial head fractures with elbow dislocation. The Mason classification system is helpful in understanding the severity of fractures and determining the appropriate management options. For instance, Mason type I fractures are relatively mild and are often treated with non-operative management, whereas type III and IV fractures tend to be more severe and best treated with surgery [4].

Using operative versus non-operative treatment for Mason type II fractures remains especially controversial. Several systematic reviews analyzing treatments for these fractures noted similar outcomes and found that there is insufficient evidence to conclude a more clinically optimal treatment option [5-6]. Additionally, recent randomized control trials utilizing Disabilities of Arm, Shoulder, and Hand (DASH) scores and Oxford Elbow Scores (OES) as primary outcome measurements reported that similar outcomes were achieved in both operative and non-operative treatment groups for Mason type II fractures, suggesting that surgery may not necessarily be indicated [7-8]. However, it remains unknown whether there are specific fracture patterns or patient characteristics that would particularly benefit from non-operative vs. operative treatment. Further studies analyzing functional outcomes of non-operative treatment for radial head fractures, would therefore provide more evidence needed to help determine the appropriate course of management.

Many patient-reported outcome measurements have been used to evaluate upper extremity injuries, including the DASH score, the OES, the American Shoulder and Elbow Surgeon score (ASES), and the Patient-Reported Outcomes Measurement Information System (PROMIS). However, notable disagreement in the categorical rankings between several of these has been shown [9]. It was further suggested that systems with several domains, those that are not reduced into a single categorical ranking, may be beneficial for clinical decision-making. PROMIS scores not only offer several domains, but they have also been shown to improve the coverage of the relevant health domains, increase reliability, and reduce test stress burdens [10-12]. There are a few, if any, studies that have utilized PROMIS scores to understand functional outcomes of non-operative treatment for radial head fractures. Hence, the current study aims to use PROMIS physical function (PF), upper extremity (UE), and pain interference (PI) domains specifically to analyze functional outcomes of non-operative management for patients with radial head fractures. We hypothesize that PROMIS PF, UE, and PI will improve throughout treatment and patients will have a corresponding positive Patient Acceptable Symptom State (PASS) score prior to the final visit.

## Materials and methods

The study was approved by the University of Rochester Institutional Review Board (IRB). A waiver of consent was granted by our IRB, as this was a retrospective evaluation of a prospectively collected database at a single, urban Level 1 trauma center. PROMIS PF (v1.2/2.0), PI (v1.1), UE (v2.0), Depression (v1.0), and Patient Acceptable Symptom State (PASS v1.0) instruments using computer adaptive test (CAT) were collected at routine clinic visits between October 1, 2015 and March 1, 2022 on Apple iPads/tablets.

Patients included in this study were identified utilizing current procedural terminology (CPT) code 24650, as well as the International Classification of Disease (ICD-10) code S52.1X. Charts were reviewed to ensure they were diagnosed with a radial head fracture based on history, physical exam, and radiographic confirmation, and underwent non-operative intervention. Inclusion criteria were patients ages 18 to 75 years, who had non-operatively treated radial head fractures between October 2015 and March 2022. We included only Mason Type I fractures and excluded types II-IV. Mason classification was determined by an orthopedic resident physician review. Additional inclusion criteria included PROMIS data from a minimum of six-weeks of follow-up. Exclusion criteria were ages outside the above range, patients with multiple injuries (i.e. polytrauma), previous elbow injury or instability, a history of impaired elbow function at baseline, and current or previous surgical intervention at the elbow. The data was de-identified and securely stored within the hospital network. 

Statistical analysis was performed with Microsoft Excel ® (Redmond, Washington) and RStudio (R Foundation for Statistical Computing, Version 2022.07.0; Vienna, Austria) Descriptive statistics including mean, standard deviation (SD), and frequency were calculated for all demographic variables. The SD and mean were calculated for every PROMIS domain evaluated at each study time point. The paired t-test was used to compare PROMIS means of the same cohort between different time points. Unpaired t-test and Chi-squared test were used to compare demographic variables. The Cohen SRM, calculated as the difference in the pre-treatment and post-treatment means, divided by the standard deviation of the difference, is an effect size index used to gauge an instrument’s responsiveness, which is defined as an instrument’s sensitivity to change over time. Using the definition of Cohen’s effect size, 0.2-0.49 is considered a small response, 0.5-0.79 is a moderate response, and 0.8 or greater is a large response. Values of p < 0.05 were considered statistically significant.

## Results

We identified 2,322 patients who sustained radial head fractures during a seven-year window for which PROMIS data is available. In total, we included 527 patients with Mason Type I radial head fractures who met the criteria for inclusion. Descriptive characteristics of the included patients are reported in Table 1. Among this cohort, 337 patients (63.9%) completed the PROMIS questionnaire at the initial post-injury office visit and 168 (31.9%) completed it at six months. Among the entire cohort, the PASS question was completed by 306 patients (58.1%) at the initial visit and 142 (26.9%) at six months. Across all patients, the average age was 48 years old (SD: 9 years), 53% of patients were female (n = 279), and the average BMI was 31 kg/m^2^ (SD: 7) (Table 1).

**Table 1:** Baseline characteristics of Mason Type I radial head fractures treated non-operatively

Characteristic	n(%) or mean (SD)
Age, years	48 (9)
BMI	31 (7)
Sex	
Female	279 (53%)
Male	248 (47%)
Race	
White	437 (83%)
Black	63 (12%)
Other	27 (5%)
Ethnicity	
Not Hispanic	480 (91%)
Hispanic	16 (3%)
Unknown	31 (6%)
Affected Side	
Left	169 (32%)
Right	358 (68%)

**Figure 1:**
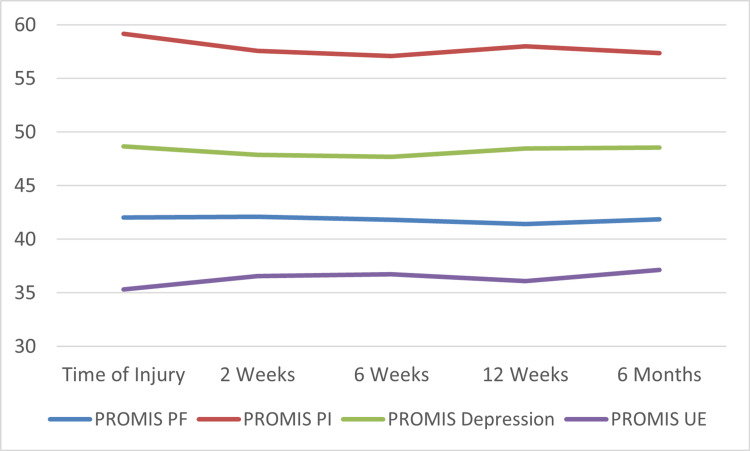
All PROMIS scores at follow-up Patient-Reported Outcomes Measurement Information System (PROMIS) Physical Function (PF), Upper Extremity (UE), Pain Interference (PI), and Depression Scores at follow-up.

At the initial outpatient post-injury visit (within one week of injury), the average PROMIS PF, UE, PI, and Depression were 42.04 (SD: 6.3), 35.31 (SD: 7.3), 59.18 (SD: 9.2), and 48.68 (SD: 6.8), respectively. At this time, among the 311 patients who completed the PASS question, 51 (16.4%) reported an acceptable symptom state. At the six-week post-injury visit, the average PROMIS PF, PI, Depression, and UE scores were 41.81 (SD: 5.4), 36.74 (SD: 7.1), 57.08 (SD: 9.2), and 47.68 (SD: 8.2) respectively. At the six-month post-injury visit, the average scores were 41.85 (SD: 7.1), 37.16 (SD: 6.2), 57.34 (SD: 9.7), and 48.55 (SD: 8.1) respectively. The recovery trajectory, as measured by PROMIS PF, UE, PI, and Depression is shown in Figure 1.

The average change in PROMIS PF, UE, PI, and Depression scores from the time of injury to six weeks were -0.23 (p=0.7), 1.43 (p=0.03), -2.1 (p=0.01), and -0.99 (p=0.1). The average change in PROMIS PF, UE, PI, and Depression scores from the time of injury to six months was -0.56 (p=0.56), 1.84 (p<0.001), -1.84 (p<0.001), and -0.13 (p=0.68). The complete PROMIS data at all follow-up time points are summarized in Table 2.

**Table 2:** All PROMIS scores at follow-up Patient-Reported Outcomes Measurement Information System (PROMIS) Physical Function (PF), Upper Extremity (UE), Pain Interference (PI), and Depression scores at follow-up.

	PROMIS PF	PROMIS UE	PROMIS PI	PROMIS Depression
Time of Injury	42.04	35.31	59.18	48.68
2 Weeks	42.09	36.56	57.56	47.87
6 Weeks	41.81	36.74	57.08	47.69
12 Weeks	41.42	36.09	58.01	48.47
6 Months	41.85	37.16	57.34	48.55
Change (6 months - initial)	-0.18	1.84	-1.84	-0.13
Significance	p=0.56	p<0.001	p<0.001	p=0.68

PROMIS UE and PI demonstrated statistically significant improvement at both six-week and six-month follow-ups (p<0.05). PROMIS PF and Depression were not significantly different between the initial visit and all follow-up time points. There was an absolute decrease in the PROMIS PF scores. PROMIS scores can be affected by other conditions and it is possible that accounts for the decrease between six-week and six-month time points. The standard response means for PROMIS PF, UE, PI, and Depression at six months were 0.03, 0.27, 0.19, and 0.02 respectively, which are classified as small responses. This data is summarized for six-week and six-month time points in Table 3.

**Table 3:** Results from PROMIS scores with six-week and six-month standard response mean Results from PROMIS Physical Function, PROMIS Upper Extremity, PROMIS Pain Interference, and PROMIS Depression with six-week and six-month Standard Response Mean.

Outcome Measure	Initial Visit Mean (SD)	6 Weeks Mean (SD)	6 Week SRM	6 Months Mean (SD)	6 Months SRM
PROMIS Physical Function	42.04 (6.3)	41.81 (5.4)	0.04	41.85 (7.1)	0.03
PROMIS Upper Extremity	35.31 (7.3)	36.74 (7.1)	0.19	37.16 (6.2)	0.27
PROMIS Pain Interference	59.18 (9.2)	57.08 (9.2)	0.23	57.34 (9.7)	0.19
PROMIS Depression	48.68 (6.8)	47.69 (8.2)	0.13	48.55(8.1)	0.02

At the six-month visit, among the 142 patients who completed the PASS question, 70 (49.3%) reported an acceptable symptom state. Among patients initially reporting “not acceptable” on PASS and reporting “acceptable” at the six-month visit, the average PROMIS PF, UE, PI, and Depression scores were 42.14, 38.91, 56.91, and 47.51 respectively. This represents an average difference of 1.11 (p=0.07), 2.82 (p<0.01), -1.19 (p=0.04), and -1.7 (p=0.01) respectively. Neither PROMIS PF, UE, PI, or Depression demonstrated a clinically significant change at six months, compared to the time of initial injury based on MCID using 1/2 standard deviation. Additionally, PROMIS UE was the only measure that showed a significant difference between PASS acceptable and not-acceptable at the initial visit and six-month follow-up. The complete PASS versus PROMIS data can be found in Table 4.

**Table 4:** PROMIS scores among patients completing PASS Patient-Reported Outcomes Measurement Information System (PROMIS) Physical Function (PF), PROMIS Upper Extremity (UE), PROMIS Pain Interference (PI), PROMIS Depression among patients completing Patient Acceptable Symptom State (PASS).

	Initial Visit				6 Month Visit (difference)			
PASS Question	PROMIS PF	PROMIS UE	PROMIS PI	PROMIS Depression	PROMIS PF	PROMIS UE	PROMIS PI	PROMIS Depression
PASS – Acceptable	43.12	34.31	59.2	47.9	42.14 (-0.98)	38.91 (4.6)	56.91 (-2.29)	47.51 (-0.39)
PASS – Not Acceptable	41.03	36.09	58.1	49.21	40.98 (-0.05)	36.98 (0.89)	57.45 (-0.65)	49.02 (-0.19)
PASS Acceptable – Not Acceptable	2.09 (p<0.01)	-1.78 (p=0.03)	1.1 (p=0.4)	-1.31 (p=0.1)	1.16 (p=0.3)	1.93 (p=0.01)	-0.54 (p=0.5)	-1.51 (p=0.04)

## Discussion

Similar short and long-term patient-reported outcome studies for Mason Type I radial head fractures have shown favorable outcomes [13-15]. Historic patient-reported outcomes used to evaluate elbow trauma include the Disabilities of the Arm, Shoulder and Hand (DASH), Short Form-36 Health Survey (SF-36), the Oxford elbow questionnaire, and patient satisfaction questionnaires. Among these tools, DASH and the Oxford elbow score were found to be correlated with objective physical exam measurements of improvement, including range of motion and strength [13-15].

PROMIS has been well established in the evaluation of other extremity conditions, including carpal tunnel syndrome, distal biceps tendon repair, elbow ulnar collateral ligament reconstruction, and elbow arthroscopy [16]. PROMIS PF and UE, specifically, have been shown to be correlated with SF-36 and DASH scores. It was noted, however, that PROMUS UE had a notable ceiling effect in younger, higher-functioning patients, which comprise the majority of this study population [16].

Given that only PROMIS UE and PI showed a significant difference at short-term and intermediate-term follow-up, it is possible that PROMIS PF and Depression are not sensitive enough to detect an improvement for non-operative management of radial head fractures, despite the success with other conditions. This could be due to the aforementioned ceiling effect or possibly because it does not ask questions that assess recovery and improvement of function for this condition. This shortcoming of PROMIS has been previously noted and may be highlighted in a traditionally non-surgical injury where patients are sometimes seen weeks after the injury [17]. This disparity might also be due to a clinically important factor that is not being captured by the PROMIS metrics, including factors such as sleep interference.

PROMIS UE and PI did demonstrate statistically significant improvement at six-week and six-month follow-up which correlated with PASS. The difference was not clinically significant as determined by the mean clinically important difference (MCID) (1/2 SD) but the correlation with PASS may better determine the clinical significance. This might be explained by the fact that patients were seen often times 10-14 days after injury when they answered the PROMIS questions for the first time. An interesting observation is the fact that 16.4% of patients reported an acceptable symptom state on PASS at their initial visit within one week of injury. This might suggest that patients felt subjectively better once they were properly immobilized or didn’t realize the extent of the injury and the function they had lost. A high positive response on PASS at the initial visit might correspond to higher baseline PROMIS scores. The improvement in PROMIS therefore might not be as significant as if they were administered immediately at the time of injury. Having a baseline PROMIS score prior to injury would aid in this analysis, however, this data was not available in our retrospective study.

Our study has several notable limitations. Due to the retrospective nature of this project and the relatively variable questionnaire response rate, PROMIS and PASS data were unavailable at some post-injury time points. There was a considerable decrease in questionnaire response rate between initial final visits, therefore, decreasing the power of long-term analysis, but likely reflective of the trauma population. Our patients were from an urban level I trauma center in the Northeast United States and might not be reflective of other geographic locations. We have evaluated the subjective outcomes based on PROMIS and PASS but have not compared them to other legacy patient-reported outcomes or objective physical exam measurements. We hypothesize that patients who have returned to their baseline or an acceptable level of function may not continue to follow up in the clinic. This may cause a selection bias, in that those who continued to follow up tended to have worse outcomes.

## Conclusions

In this study, we found: (1) PROMIS UE and PI significantly improved among Mason I radial head fractures treated non-operatively at both six-week and six-month follow-up points but did not meet MCID; (2) PROMIS PF and Depression did not significantly differ between the time of injury, six-week or six-month follow-up points; (3) Only PROMIS UE correlated with PASS at six-week and six-month follow-ups; (4) Among patients who improved from negative to positive response on PASS, PROMIS UE, PI, and Depression significantly improved. This insight provides a framework for discussion about the utility of PROMIS in evaluating outcomes of radial head fractures despite the success of PROMIS with other conditions. The results from this study suggest that PROMIS UE and PI may be able to detect a change that is statistically significant amongst this population but fails to capture a clinically significant difference based on MCID.
